# A worldwide bibliometric analysis of acromegaly in the past two decades: 1999–2022

**DOI:** 10.3389/fnins.2023.1187820

**Published:** 2023-07-05

**Authors:** Shuqin Peng, Qi Liu, Yuanyuan Teng, Biling Huang, Ze Liu, Mingliu Li, Jieyu Liang, Yi Zhang, Min Wang

**Affiliations:** ^1^Department of Endocrinology, Xiangya Hospital, Central South University, Changsha, Hunan, China; ^2^National Clinical Research Center for Geriatric Disorders, Xiangya Hospital, Central South University, Changsha, Hunan, China; ^3^Department of Orthopaedics, Xiangya Hospital, Central South University, Changsha, Hunan, China

**Keywords:** acromegaly, bibliometrics analysis, visual analysis, Web of Science, CiteSpace, VOSviewer

## Abstract

**Objectives:**

To conduct a bibliometric analysis to quantify and identify the current status and trends of acromegaly research in the past two decades.

**Materials and methods:**

Articles related to acromegaly that were published from 1999 to 2022 were retrieved through the Web of Science core collection (WoSCC) database. Then, they were imported into VOSviewer and CiteSpace to conduct a visualization analysis of authors, countries, institutions, citation numbers, cocitations, keywords, and references.

**Results:**

A total of 3,909 articles were identified in the study. Among them, the United States made the largest contribution to the field. Moreover, Colao A. was the most prolific author, and the University of Naples Federico II was the institution with the most publications. In addition, the *Journal of Clinical Endocrinology and Metabolism* was the core journal in the field. High-frequency keywords mainly included “acromegaly,” “GH (Growth Hormone),” “IGH-I (Insulin-Like Growth Factor I),” “pituitary adenomas,” and “octreotide.”

**Conclusion:**

Studies related to acromegaly have shown stable stepwise growth over the past two decades. Interestingly, the research focus after 2016 gradually shifted from the etiology, mechanism, medications for treatment, and complications to improving prognosis and quality of life of patients with acromegaly. The current findings may provide guidance for further research in the field of acromegaly.

## 1. Introduction

Acromegaly is a severe and chronic endocrine disease characterized by excessive secretion of growth hormone (GH) and insulin-like growth factor I (IGF-I) (Melmed, 2020). In the majority of cases, this excessive secretion is induced by a GH-secreting pituitary tumor; however, in rare cases, it is induced by pituitary hyperplasia or ectopic secretion of GH or GH-releasing hormone (GHRH) (Petrossians et al., 2017). Both gigantism and acromegaly are rare disorders that are caused by excessive secretion of GH and IGF1, and acromegaly occurs when excess GH is present in individuals after epiphyseal closure (Hannah-Shmouni et al., 2016). At the time of diagnosis, patients usually present with acral overgrowth including exaggerated growth of the hands and feet, facial overgrowth including prognathism (a protruding jaw), and soft-tissue hypertrophy (Colao et al., 2004; Katznelson et al., 2014; Hannah-Shmouni et al., 2016; Petrossians et al., 2017). According to the first systematic review and meta-analysis of epidemiologic studies of acromegaly, which was published in 2021, the pooled global prevalence of acromegaly is 5.9 (95% CI: 4.4–7 0.9) per 100,000 persons and the pooled global incidence rate is 0.38 (95% CI, 0.32–0.44) per 100,000 person-years (Crisafulli et al., 2021).

The term “acromegaly” was coined in 1886 by the French neurologist Pierre Marie to describe the characteristic clinical features of a woman with the disease (Marie, 1886). Many achievements in acromegaly have been made over the past 100 years, such as medical treatment and the availability of novel radiotherapy techniques in acromegaly (Colao et al., 2019). However, we still lack a grasp of the current overall situation regarding acromegaly. Although a quantitative overview could be conducted by many approaches, bibliometrics can qualitatively and quantitatively analyze the contribution and cooperation of authors, institutions, countries, and journals and, at the same time, evaluate the knowledge base and trending research topics (Chen and Song, 2019; Zhang et al., 2021).

Therefore, our study aims to use two bibliometric software programs, namely, CiteSpace and VOSviewer, to quantify the current status of acromegaly research and to identify the trending research questions of the past two decades. These findings could help to generate hypotheses for future studies in the field of acromegaly.

## 2. Materials and methods

### 2.1. Data sources and search strategy

**Figure 1:**
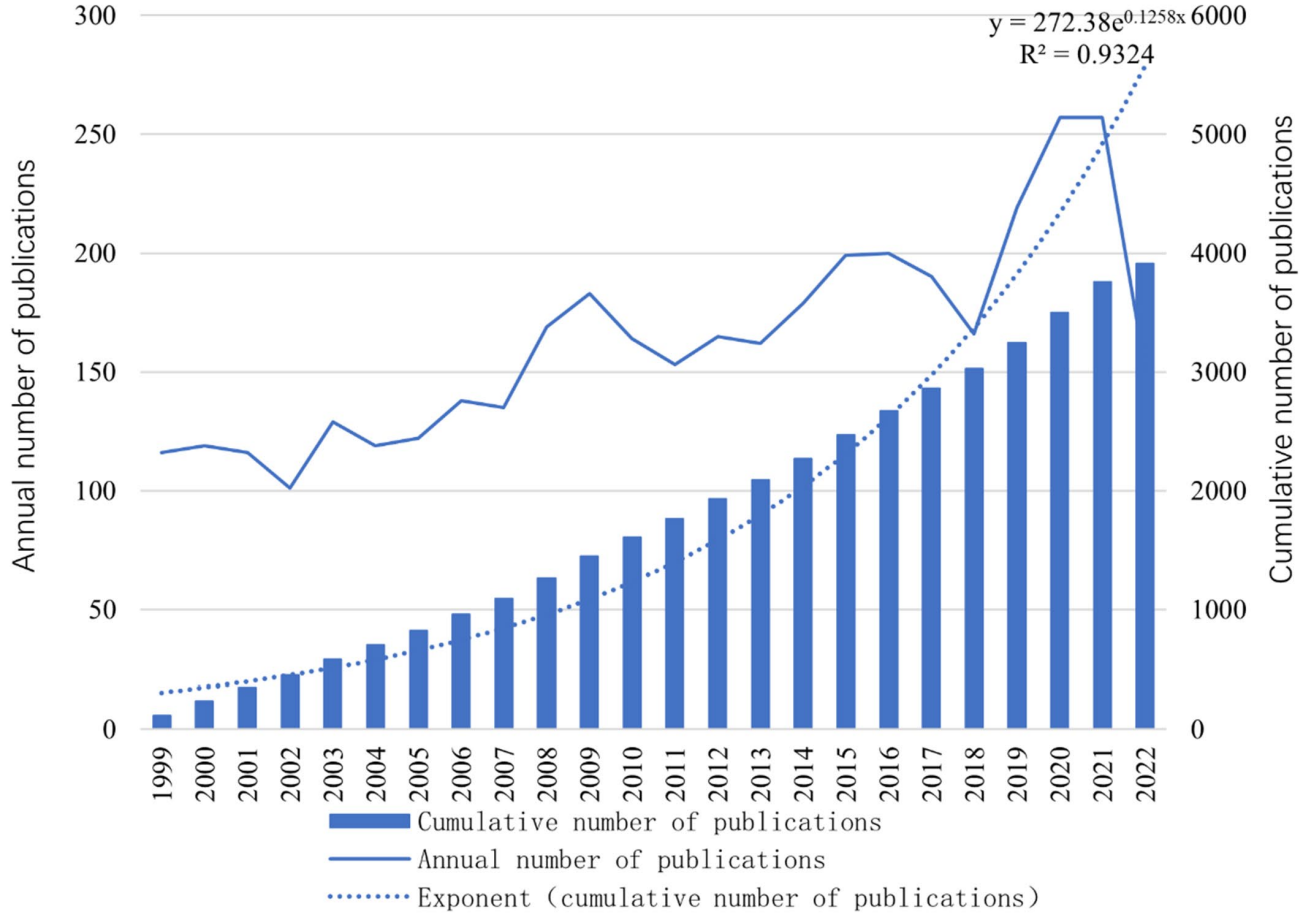
The annual trends in the publications on acromegaly from 1999 to 2022.

The data were retrieved from the Web of Science Core Collection (WoSCC) Database, and the search strategy is listed as follows: topic search (TS, the topic search scope includes the fields title, abstract, author keywords, and keywords plus) = (“Acromegaly”) (Figure 1). Only studies that were published from 1999 to 2022 were eligible for inclusion. To avoid potential bias, the search was performed on 22 October 2022, and only studies published in English were included. Review and articles were eligible for inclusion. The search results were downloaded as “Full Record and Cited References” and “Plain Text.” For further analysis, we subsequently renamed the files “download_*.txt,” which CiteSpace software could read.

### 2.2. Data analysis

CiteSpace 6.1.3 (64-bit) basic, VOSviewer 1.6.18, and Microsoft Excel 2022 were used for bibliometric analysis. The first step was to clean the data. For example, “growth hormone” and “growth-hormone” were merged as “GH,” “growth-factor-I,” “factor-I,” “igf-1,” “insulin-like growth factor I,” and “insulin-like growth factor-1” were merged as “IGH-I,” and “pituitary-adenoma” was identified as “pituitary adenomas” (Paunkov et al., 2019).

CiteSpace was used to analyze the co-occurrence of countries/regions and institutions, the dual-map of journals, reference timelines, citation bursts, keyword timelines, and keyword bursts. The settings were as follows: timespan (1999–2022), years per slice (1), scale factor *k* = 15, and selection criteria (Top *N* = 50). Cluster labels were extracted by the log-likelihood ratio (LLR) algorithm. Other parameter settings followed the initial software settings.

VOSviewer was used to perform a visual analysis of authors, institutions, and countries. In the cluster map, the size of the node reflects the co-occurrence frequencies, the link indicates the co-occurrence relationship, and the thickness of the link is proportional to the number of times two keywords co-occur. In the visual map, the node size represents the co-occurrence frequency, the links represent the co-occurrence relationship, and the node color represents its affiliation with different clusters. In the overlay map, the node size represents the co-occurrence frequency, the links represent the co-occurrence relationship, and the node color represents the timeline.

Excel software was used to analyze the annual publications. We obtained the impact factor (IF) and Journal Citation Reports (JCR) division of journals from the Web of Science on 22 October 2022.

## 3. Results

### 3.1. Analysis of article numbers and trends

We retrieved 4,068 research papers from the WoSCC database; ultimately, 3,909 eligible articles were included. As shown in Figure 1, the number of articles on acromegaly has steadily increased over the past two decades.

### 3.2. Analysis of national cooperation

Bibliometric analysis revealed that the included articles on acromegaly were published in 80 countries/regions between 1999 and 2022. The top 10 contribution countries are presented in Table 1. The United States contributed the most articles (*n* = 828/21.18%), followed by Italy (*n* = 612/15.66%) and England (*n* = 375/9.59%). The national cooperation visualization map shows that close cooperation between countries/regions around the world was an extremely common phenomenon. The top 49 countries/regions according to the number of publications (*n* ≥ 50) on acromegaly are presented in Supplementary Figure S1, which indicates that the United States had the most instances of cooperation, and it has established close cooperation with several countries such as Italy. Additionally, China and Turkey have recently published a large number of articles.

**Table 1:** The top 10 countries/regions that contributed to acromegaly research.

Rank	Country/Region	Articles	Count (%)
1	USA	828	21.18%
2	Italy	612	15.66%
3	England	375	9.59%
4	Germany	300	7.67%
5	Türkiye	286	7.32%
6	Japan	274	7.01%
7	Netherlands	253	6.47%
8	France	245	6.27%
9	Brazil	242	6.19%
10	Spain	219	5.60%

### 3.3. Analysis of authors

Table 2 lists the top 10 most productive authors. These 10 authors published a total of 565 articles, accounting for 14.45% of all included articles. Colao A. (*n* = 120), from the University of Naples Federico II in Italy, had the most publications, followed by Chanson P. (*n* = 66) from the University Paris-Saclay and Giustina A. (*n* = 54) from San Raffaele University Hospital, Italy. The articles published by Colao A. had the highest number of citations (*n* = 6,869) and Melmed S. had the highest number of average citations per article (108.11), which means their studies have attracted the highest amount of attention from other scholars. Furthermore, the overlay visualization map of the co-cited authors was also generated by VOSviewer software and is shown in Supplementary Figure S2, with the color of the nodes indicating the average publication year of the author’s articles. Xing B. and Wang RZ. et al. from China made great contributions to acromegaly research in 2020, which indicates that research on acromegaly is developing rapidly in China.

**Table 2:** The top 10 most productive authors involved in acromegaly research.

Rank	Author	Articles	Citations	Citations per article
1	Colao A.	120	6,869	57.24
2	Chanson P.	66	3,390	51.36
3	Giustina A.	54	3,411	63.17
4	Gadelha MR.	53	1,501	28.32
5	Lombardi G.	50	4,346	86.92
6	Fleseriu M.	49	1,281	26.14
7	Pivonello R.	46	1828	39.74
8	Melmed S.	44	4,757	108.11
9	Korbonits M.	42	1,276	30.38
10	Biermasz NR.	41	1,160	28.29

### 3.4. Analysis of institutional cooperation

A total of 3,587 institutions participated in acromegaly research. As shown in Table 3, the University of Naples Federico II was the most productive institution, with 129 research papers published (3.30%), followed by Leiden University (*n* = 90; 2.30%) and the Federal University of Rio de Janeiro (*n* = 87; 2.23%). The collaboration network was generated by VOSviewer software, and the threshold was set to 20 as the minimum number of articles for each institution. Ultimately, 85 out of the 3,587 institutions were identified (Supplementary Figure S3), and the node color represents its affiliation with different co-occurrence clusters. As shown in the figure, the University of Naples Federico II and the University of Brescia established close cooperation. These institutions have made significant contributions to the field. The cooperation between these top institutions in the same country has greatly advanced the field, but it also limits international communication and cooperation. Therefore, transnational cooperation should be further strengthened and encouraged.

**Table 3:** The top 10 organizations involved in acromegaly research.

Rank	Organization	Articles	Count (%)	Citations	Country
1	University of Naples Federico II	129	3.30%	8,295	Italy
2	Leiden University	90	2.30%	3,846	Netherlands
3	Federal University of Rio de Janeiro	87	2.23%	2,753	Brazil
4	University of Milan	80	2.05%	3,459	Italy
5	University of Brescia	77	1.97%	6,238	Italy
6	University of Turin	68	1.74%	2,840	Italy
7	Cedars Sinai Medical Center	66	1.69%	6,882	USA
8	Oregon Health and Science University	64	1.64%	2,565	USA
9	University of Virginia	63	1.61%	4,757	USA
10	University of Genoa	62	1.59%	2,701	Italy

### 3.5. Analysis of journals

**Figure 2:**
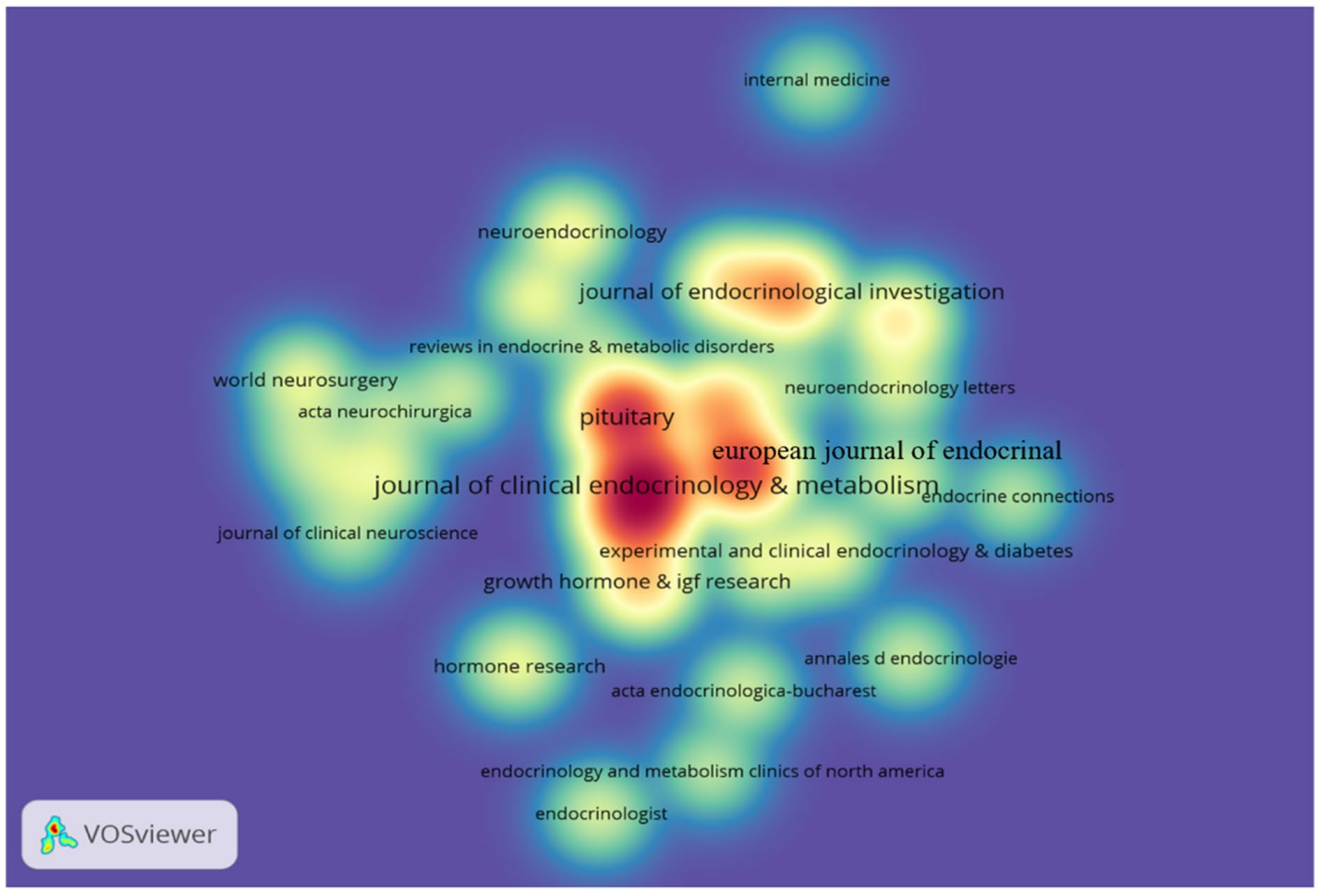
VOSviewer density visualization map of cited journals. The size of the word, the size of the roundish shape, and the opacity of the yellow are positively related to the cited frequency. The minimum citation number is 20.

A total of 701 academic journals published articles on acromegaly research, and a comprehensive analysis of the contribution of journals was conducted, including journal characteristics such as journal titles, articles number, articles count, total citations, region, IF (2021), and JCR division (Table 4). The top 10 journals published a total of 1,803 research papers, accounting for 46.12% of all included publications. *The Journal of Clinical Endocrinology and Metabolism* had the greatest number of publications (*n* = 374; 9.57%), followed by *Pituitary* (*n* = 305; 7.80%) and *European Journal of Endocrinology* (*n* = 274; 7.01%). The density visualization map of cited journals in Figure 2 provides a more intuitive illustration of this data.

**Table 4:** The top 10 journals involved in acromegaly research.

Rank	Source	Articles	Count (%)	Citations	Region	IF (2021)	JCR division
1	Journal of Clinical Endocrinology and Metabolism	374	9.57%	26,214	USA	6.134	Q1
2	Pituitary	305	7.80%	6,664	USA	3.599	Q3
3	European Journal of Endocrinology	274	7.01%	10,062	England	6.558	Q1
4	Clinical Endocrinology	235	6.01%	10,310	England	3.523	Q3
5	Journal of Endocrinological Investigation	152	3.89%	2,353	Italy	5.467	Q2
6	Endocrine	135	3.45%	1903	USA	3.925	Q3
7	Growth Hormone and IGF Research	111	2.84%	1,575	England	2.125	Q4
8	Endocrine Journal	90	2.30%	1,011	Japan	2.86	Q4
9	Frontiers in Endocrinology	78	2.00%	521	Switzerland	6.055	Q1
10	Neuroendocrinology	49	1.25%	1,183	Switzerland	5.135	Q2

### 3.6. Co-cited references and reference burst

Of the 57,530 cited references, 90 references were cited at least 100 times. Table 5 shows that the top 10 co-cited references were co-cited at least 368 times.

**Table 5:** The top 10 co-cited references related to acromegaly.

Rank	Title	First author	Journal	Year	Citations
1	Systemic complications of acromegaly: epidemiology, pathogenesis, and management	Colao A.	Endocrine Reviews	2004	629
2	Acromegaly: an endocrine society clinical practice guideline	Katznelson L.	J. Clin. Endocrinol. Metab.	2014	621
3	Criteria for cure of acromegaly: A consensus statement	Giustina A.	J. Clin. Endocrinol. Metab.	2000	596
4	Criteria for cure of acromegaly: A consensus statement	Melmed S.	J. Clin. Endocrinol. Metab.	2006	556
5	A Consensus on Criteria for Cure of Acromegaly	Giustina A.	J. Clin. Endocrinol. Metab.	2010	426
6	Mortality and cancer incidence in acromegaly: a retrospective cohort study. United Kingdom Acromegaly Study Group.	Orme SM.	J. Clin. Endocrinol. Metab.	1998	409
7	Treatment of acromegaly	Holdaway IM.	J. Clin. Endocrinol. Metab.	2004	374
8	Guidelines for Acromegaly Management: An Update	Melmed S.	J. Clin. Endocrinol. Metab.	2009	373
9	Long-term mortality after transsphenoidal surgery and adjunctive therapy for acromegaly.	Swearingen B.	J. Clin. Endocrinol. Metab.	1998	370
10	Treatment of acromegaly with the growth hormone-receptor antagonist pegvisomant	Trainer PJ.	New England Journal of Medicine	2000	368

**Figure 3:**
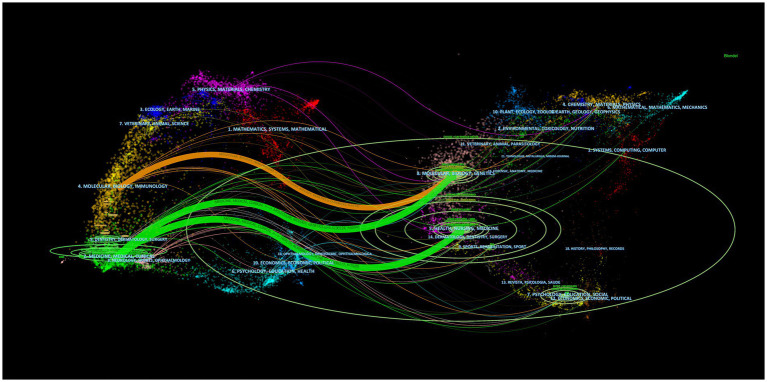
The dual-map overlay of journals stands for the topic distribution of academic journals. The citing journals are on the left, the cited journals are on the right, and the colored path represents the citation relationship.

The dual-map overlay of journals depicts the distribution of topics in scientific journals (Figure 3). Citing and cited journals are located on the left and right, respectively, and the colored path represents the citation relationship. It can be seen that three major paths were observed between the citing and the cited journals. The strongest citation relationships were from Medicine/Medical/Clinical journals to Health/Nursing/Medicine journals.

**Figure 4:**
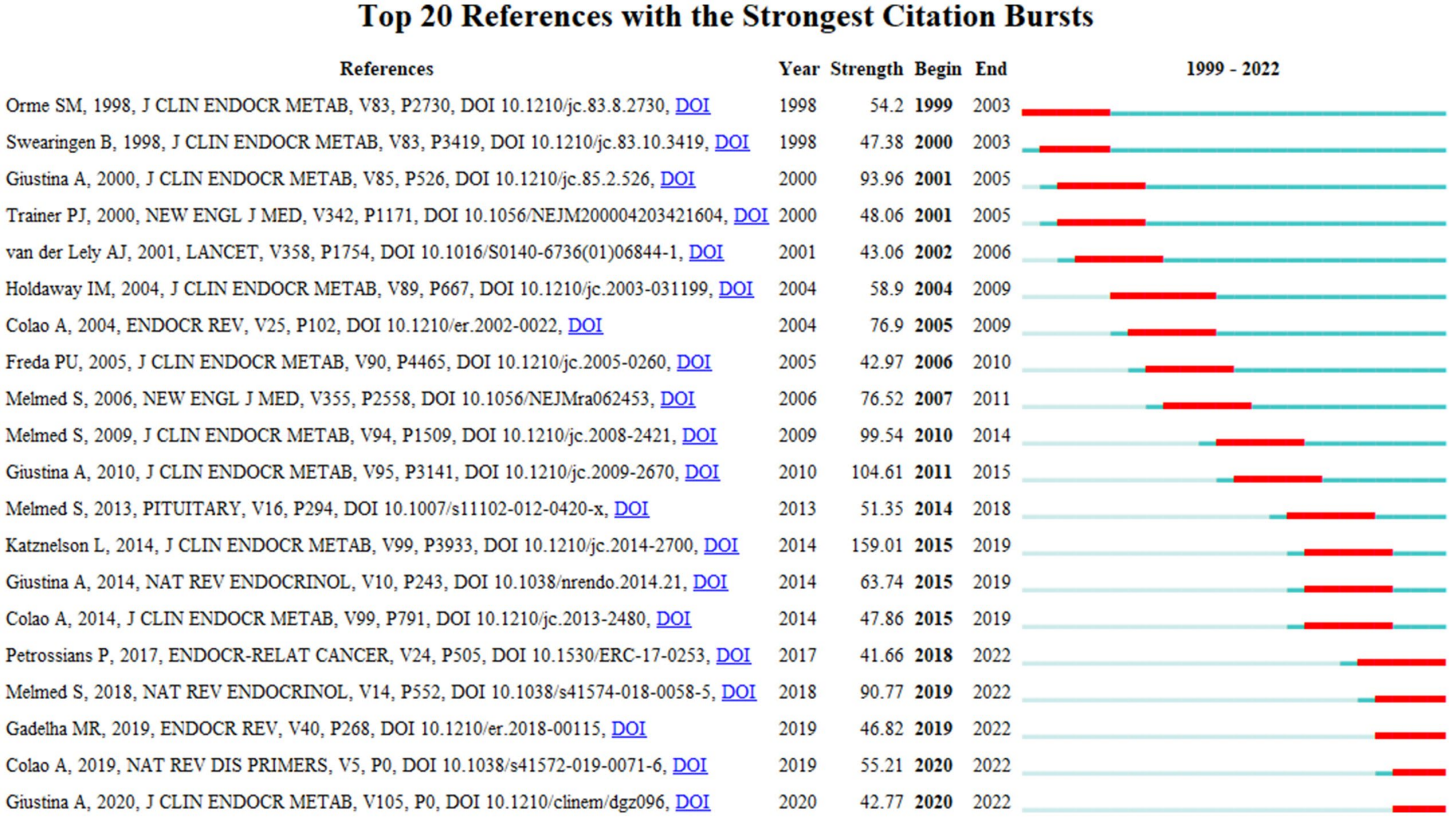
The top 20 references with the strongest citation bursts involved in acromegaly research from 1999 to 2022. Years in light green mean that the reference has not yet appeared, years in dark green mean that the reference is less influential, and years in red mean that the reference is more influential.

References with citation bursts are those that have been cited significantly more frequently over a period (Chen, 2017). A total of 287 references were identified as citation bursts, and we listed the top 20 in Figure 4. The earliest burstiness (strength = 54.2) occurred in a study entitled “Acromegaly: An Endocrine Society Clinical Practice Guideline” (Orme et al., 1998), published in the *Journal of Clinical Endocrinology and Metabolism* by Orme SM. et al. in 1999, with citation business from 1999 to 2003. The strongest burstiness (strength = 159.01) occurred in a study entitled “Acromegaly: an endocrine society clinical practice guideline” (Katznelson et al., 2014), published in the *Journal of Clinical Endocrinology and Metabolism* by Katznelson L. et al. in 2014, with citation burstiness from 2015 to 2019. In addition, five references (Petrossians et al., 2017; Melmed et al., 2018; Colao et al., 2019; Gadelha et al., 2019; Giustina et al., 2020a,b) Are still exhibiting burstiness. These articles have focused on the disease pathogenesis, diagnosis, complications, new medical therapies, and management of acromegaly. In these articles, acromegaly has been examined in detail from various perspectives. It is worth noting that the article by Petrossians P. et al., which was published in 2017, showed clinically relevant trends in the characteristics of acromegaly at diagnosis (Petrossians et al., 2017).

### 3.7. Analysis of keywords

**Figure 5:**
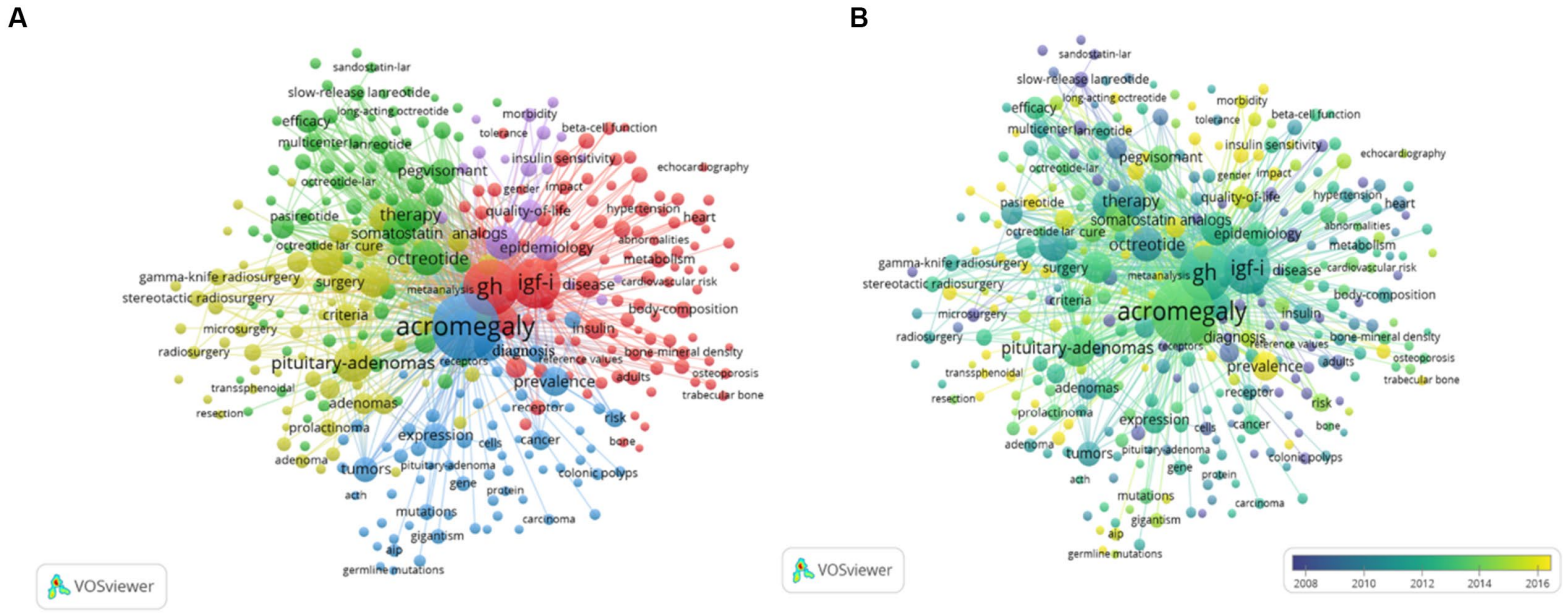
The top 338 co-occurrence keywords visual map **(A)** and the overlay map **(B)**. Keywords appeared ≥20, max lines = 1,000. The node size reflects the co-occurrence frequencies, the link indicates the co-occurrence relationship, and the thickness of the link is proportional to the number of times two keywords co-occur. **(A)** Using different colors, the 338 keywords that appeared more than 20 times were separated into five clusters: Cluster 1 (red) is primarily concerned with the molecular mechanism of acromegaly, cluster 2 (green) is primarily related to the drug treatment of acromegaly, cluster 3 (blue) is mainly about the diagnosis and epidemiology of acromegaly, cluster 4 (yellow) primarily focuses on the etiology of acromegaly, pituitary adenomas, and its surgical treatment, and cluster 5 (purple) focuses on the prognosis and quality of life of patients with acromegaly. **(B)** Keyword visualization according to the average publication year. The different colors indicate the relevant year of publication. Yellow keywords came later than blue keywords.

A total of 8,390 keywords were extracted, among which 595 keywords appeared at least 10 times and 43 keywords appeared at least 100 times, as shown in Table 6. The 10 most frequent keywords are shown in Table 6. “Acromegaly” was the most frequent keyword (*n* = 2,056), followed by “GH” (*n* = 1,307), “IGF-I” (*n* = 1,008), “pituitary adenomas” (*n* = 576), and “octreotide” (*n* = 464). The top 338 keywords (*n* ≥ 20) are also presented in the visualization map in Figure 5A. There are five colors in the figure, representing the five clusters in the keyword co-occurrence network. Cluster 1 is in red, in which the high-frequency keywords “GH” and “IGF-I” are related to the molecular mechanism of acromegaly. Cluster 2 is in green, and the high-frequency words were “octreotide,” “therapy,” and “somatostatin analogs,” which were related to medications for the treatment of acromegaly. Cluster 3 is in blue, and the high-frequency words were “acromegaly,” “diagnosis,” and “prevalence,” which were related to the diagnosis and epidemiology of acromegaly. Cluster 4 is in yellow, and the high-frequency words were “pituitary-adenomas,” “transsphenoidal surgery,” and “management,” which were related to the etiology of acromegaly, pituitary adenomas, and its surgical treatment. Cluster 5 appears in purple, and the high-frequency words were “mortality,” “epidemiology,” and “quality-of-life,” which were related to the prognosis and quality of life of patients with acromegaly.

**Table 6:** Top 10 co-occurrence keywords.

Rank	Keywords	Occurrences
1	Acromegaly	2056
2	GH	1,307
3	IGH-I	1,008
4	Pituitary adenomas	576
5	Octreotide	464
6	Mortality	442
7	Transsphenoidal surgery	407
8	Therapy	375
9	Management	374
10	Diagnosis	335

GH, Growth Hormone; IGH-I, Insulin-Like Growth Factor I.

An overlay visual graphic that can be compared to a co-occurrence is shown in Figure 5B, and the color of the item varies according to the average time of appearance of the term, with yellow indicating the nearest time. The items show that a considerable number of nodes of different colors, ranging from purple to yellow, can be observed in all five clusters, reflecting the balanced development of each of the five research fields over the past two decades. Revealingly, the cluster focused on the prognosis and quality of life of patients with acromegaly accounted for a large part of the yellow cluster after 2016.

**Figure 6:**
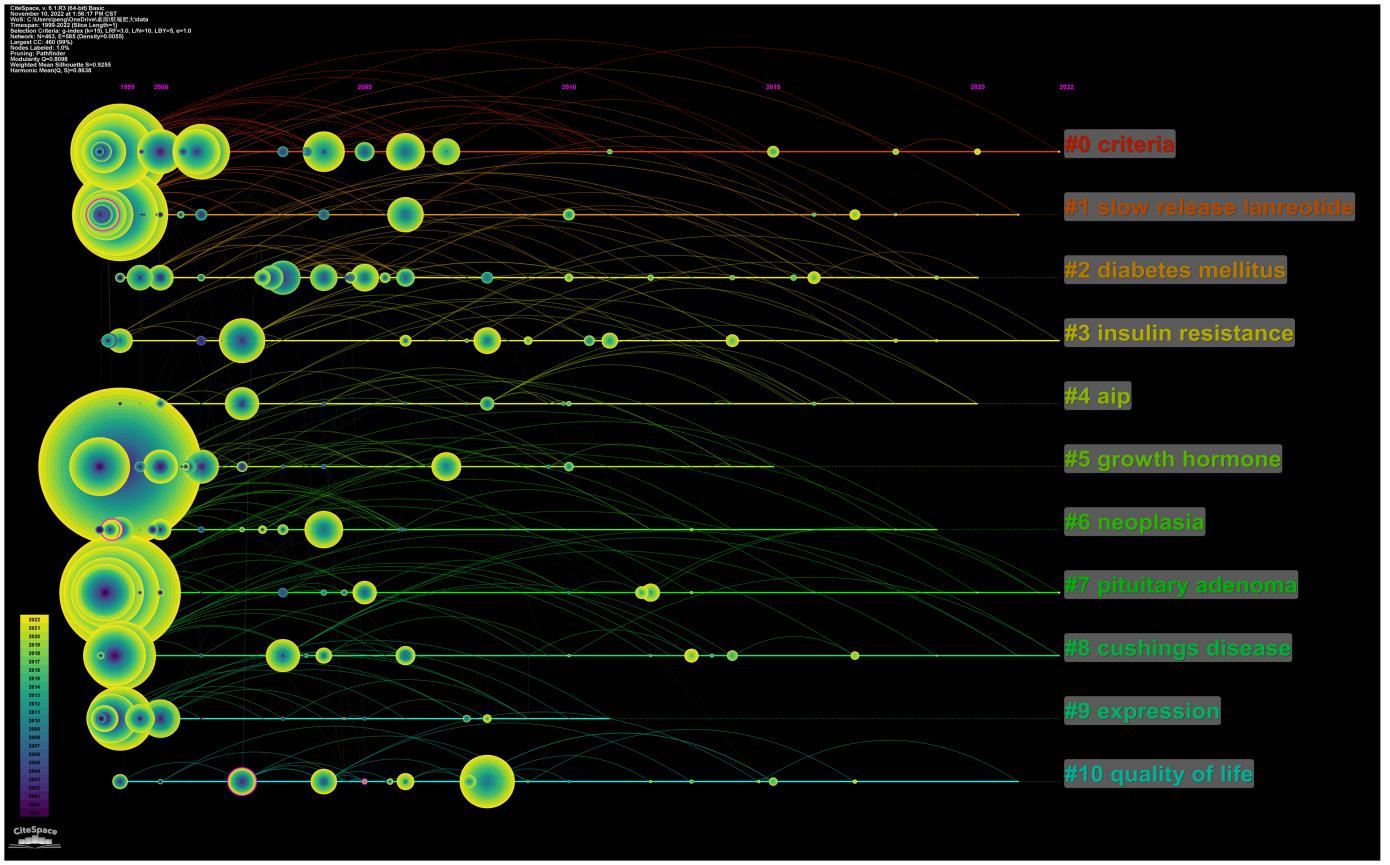
The timeline view of keywords related to acromegaly. Timeline view of keywords related to acromegaly. Each horizontal line represents a cluster; the smaller the number, the larger the cluster, and #0 is the largest cluster. The node size reflects the co-occurrence frequencies, and the links indicate the co-occurrence relationships; the colors of the node and line represent different years; nodes are at their first co-occurrence year. Cluster labels were extracted from the title by LLR.

Furthermore, the LLR algorithm in CiteSpace was used to cluster keywords. A total of 19 clusters are shown in Supplementary Figure S4. The top five keyword clusters were “criteria,” “slow release lanreotide,” “diabetes mellitus,” “insulin resistance,” and “AIP (aryl hydrocarbon receptor-interacting protein)” (Table 6). We also represented the keywords by timeline view to show the chronological changes (Figure 6). These findings suggest that the current research on acromegaly mainly focuses on the etiology, mechanism, treatment, and complications of the disease.

## 4. Discussion

A total of 3,909 articles were identified, including 14,502 authors, 3,587 institutions, 80 countries/regions, and 701 journals, and 57,530 articles from 7,952 journals were cited. The number of articles published gradually increased from 1999 to 2022, which suggests that our understanding of acromegaly is deepening and expanding.

Author Colao A. made great contributions to research on acromegaly. The most frequently cited paper (*n* = 629) was published by Colao A. et al. in 2004 and described the systemic complications of acromegaly from the three aspects of epidemiology, pathogenesis, and management. A recently published article, “Global psychological assessment with evaluation of life and sleep quality, sexual and cognitive function in a large number of patients with acromegaly: A cross-sectional study” (Pivonello et al., 2022), demonstrated that acromegaly is associated with a persisting impairment of a patient’s psychological profile despite remission and long-term medical treatment.

The articles on acromegaly were mainly published in endocrinology and metabolism journals. As the journal with the largest number of publications, *The Journal of Clinical Endocrinology and Metabolism* (Region: United States, IF = 6.134, Q1) belongs to the top journals in endocrinology and metabolism and publishes articles and reviews that focus on the clinical aspects of endocrinology, including the clinical application of molecular endocrinology. According to the 2021 Journal Citation Reports (JCR), three of the top 10 journals belong to Q1, and eight journals have an impact factor (IF) of greater than 3. In addition, as these journals are professional journals with high visibility and influence, it may be easier for scholars to publicize their ideas or views in the field of science, thus enabling them to discuss and exchange ideas with their peers, improving their academic level and scientific ability. Finally, these journals have a relatively short review cycle. Therefore, scholars prefer to submit articles to these journals. Based on this trend, the journals shown in Table 4 may continue to be the “primary conduit” for future results in the discipline. Additionally, this has prompted scholars interested in the subject to read these publications more carefully.

The collection of co-cited references cited by the corresponding research community could partly represent the knowledge base (Chen, 2017). Among the top 10 co-cited references, seven mainly described the guidelines, criteria, and consensus of several aspects of acromegaly (Giustina et al., 2000, 2010; Colao et al., 2004; Holdaway, 2004; Melmed et al., 2009; Katznelson et al., 2014; Casanueva et al., 2017), including epidemiology, pathogenesis, treatment and management of the disease. One reference was about mortality and cancer incidence in acromegaly (Orme et al., 1998), one focused on long-term mortality after transsphenoidal surgery and adjunctive therapy for acromegaly, and one was about the treatment of acromegaly with the growth hormone-receptor antagonist pegvisomant (Trainer et al., 2000). Regarding reference burst analysis, five references (Petrossians et al., 2017; Melmed et al., 2018; Colao et al., 2019; Gadelha et al., 2019; Giustina et al., 2020a,b) are still exhibiting burstiness. These articles focused on the disease pathogenesis, diagnosis, complications, new medical therapies, and management of acromegaly. In these articles, acromegaly has been examined in detail from various perspectives. It is worth noting that the article published by Petrossians P. et al. in 2017 described clinically relevant trends in the characteristics of acromegaly at diagnosis (Petrossians et al., 2017). Thus, we could identify that current research focuses on the pathogenesis, diagnosis, treatment, and management of acromegaly.

With the progress of acromegaly research, some emerging research fields have gradually become research hotspots. Figure 5A shows the keyword co-occurrence analysis. The purpose of co-occurrence was to assess links between items already recorded. It is considered a useful tool for predicting the evolution and hot spots of a particular academic topic. By analyzing the keywords in the literature, we constructed a network map of co-occurrence relationships. Five possible research directions were identified as follows (Figure 5A): “the diagnosis and epidemiology of acromegaly,” “etiology of acromegaly,” “the molecular mechanism of acromegaly,” “the medical treatment of acromegaly,” and “the prognosis and quality of life of acromegaly patients.” Based on the five abovementioned research directions, we summarized the relevant research progress. First, understanding the prevalence and diagnosis of acromegaly is helpful for disease management. The study of Aagaard C. et al. suggests that the prevalence of acromegaly significantly increased over a three-decade period (1992–2021) (R2 = 0.94, *p* < 0.001), whereas the annual incidence remained constant and the clinical presentation shifted toward a milder phenotype. In addition, previous studies have shown that the global combined prevalence of acromegaly is 5.9 (95% CI: 4.4–7.9) per 100,000 person-years (Crisafulli et al., 2021), and the mortality rate of patients with acromegaly increased by 72% compared with the general population (Dekkers et al., 2008). Screening of patients with clinical suspicion or associated comorbidities can reduce the delay in diagnosis. Accurate diagnosis requires rigorous evaluation based on biochemical, imaging, and pathological markers (Fleseriu et al., 2022). Moreover, the research by Cuevas-Ramos D. et al. shows that the classification of acromegaly may be useful to accurately identify patients with acromegaly who present with distinctive patterns of disease aggressiveness and outcome, as well as to provide an accurate tool for selection criteria in clinical studies (Cuevas-Ramos et al., 2015). Second, in terms of etiology, growth hormone-secreting pituitary adenomas are present in approximately 99% of people with acromegaly, whereas in the remaining 1% of cases, acromegaly develops due to rare ectopic tumors secreting GHRH or growth hormone, or both (Melmed, 2006; Ghazi et al., 2013). Pituitary Ectopic tumoral GHRH secretion somatotroph hyperplasia leads to a secondary tumor appearing as an enlarged pituitary gland on MRIs, often mimicking a pituitary adenoma. Primary GH hyperplasia can also be present in rare genetic syndromes. Ectopic GH produced by neuroendocrine tumors or lymphomas is very rare and MRIs may show a normal or small pituitary gland (Ghazi et al., 2013; Potorac et al., 2022). Third, in terms of molecular mechanisms, growth hormone-secreting pituitary adenomas in acromegaly arise as monoclonal expansions of differentiated somatotroph cells. Mutations in the activation of GNAS (commonly known as Gsp), which are present in up to 40% of GH (growth hormone) adenomas, inhibit GTPase activity, leading to cAMP accumulation and excess GH synthesis (Melmed et al., 2022). Substitution of Arg201 or Gln227 results in constitutive activation of the mutated Gsα subunit. Somatotroph adenomas also exhibit high levels of PDE4D expression, further maintaining cAMP accumulation. Hypothalamic or tumor-derived ectopic GHRH induces cAMP, resulting in DNA damage, GH induction, and numerous somatic copy number alterations (Ben-Shlomo et al., 2020). Thus, constitutive cAMP activation mimics excess GHRH signaling, which induces somatotroph proliferation with DNA damage and GH overproduction (Fleseriu et al., 2022). In addition, major medical treatments are typically reserved for patients who have contraindications to or refuse surgery and for those with persistent growth-hormone hypersecretion after surgery (Katznelson et al., 2014; Melmed et al., 2018; Giustina et al., 2020b). Somatostatin receptor ligand is currently the mainstay in the medical management of acromegaly and is indicated for the majority of patients as first-line medical therapy (Giustina et al., 2020b). The development of biomarkers in response to treatment and the use of these biomarkers to guide clinical decision-making will improve the management of patients with acromegaly. Therefore, the research on this class of drugs is promising. Vertebral fractures (VFs) are a serious complication of acromegaly (Biermasz et al., 2005; Claessen et al., 2016). There is a very high prevalence of VFs in acromegaly patients with long-term controlled disease (Wassenaar et al., 2011). Some studies show that pasireotide may positively influence bone metabolism in acromegaly (Giustina et al., 2008), and this may have a good effect on the prevention of VFs. Furthermore, we used a variety of tools to specifically assess prognosis and quality of life in patients with acromegaly, including AcroQoL and PASQ, and the Acromegaly Treatment Satisfaction Questionnaire (AcroTSQ) (Fleseriu et al., 2020). In addition, customized and personalized treatment plans are essential to improve the quality of life of patients with acromegaly (Fleseriu et al., 2022). Coopmans et al. (2022) suggested that a patient-centered approach should be considered when making treatment decisions, taking into consideration conventional biochemical outcomes, tumor control, comorbidities, treatment complications, and PROMs, including QoL measures. Mizera et al. (2018) showed that cardiovascular disease is the principal cause of premature mortality in patients with acromegaly, accounting for approximately 60% of deaths. Among cardiovascular risk factors, arterial hypertension was reported in 35% of patients with acromegaly. Therefore, early and aggressive hypertension treatment is essential for the prognosis of acromegaly. Despite the numerous findings in the abovementioned areas of acromegaly research, future developments in these areas are still promising.

An overlay visual graphic (Figure 5B) can instantly track research progress and predict future hot topics. The results showed that the cluster corresponding to the purple cluster in Figure 5A, which focused on the prognosis and quality of life of patients with acromegaly, accounted for a large part of the yellow items after 2016. This indicates that, after 2016, the research focus of acromegaly gradually shifted from exploring the etiology, mechanism, diagnosis, complications, medications for treatment, and epidemiology of the disease to the prognosis of patients and their overall quality of life.

## 5. Strengths and limitations

Based on the current bibliometric analysis and visualization analysis, the trends and hotspots of acromegaly research can be better understood. However, this study also has some limitations. First, the SCI extension data only included articles and reviews in English. Second, the literature only relied on the WoSCC database, and publications in other databases such as PubMed and Cochrane may not have been identified. Third, because VOSviewer and CiteSpace cannot analyze the complete text of a publication, certain information may have been missed. Finally, some recently published articles were not included in the current analyses.

## 6. Conclusion

Acromegaly research has shown stable stepwise growth in the past two decades. The etiology, mechanism, complications, medications for treatment, and epidemiology of acromegaly have always been the main research directions. However, after 2016, the research direction gradually shifted to improving the prognosis and the overall quality of life of patients with acromegaly. The current findings may provide guidance for further research in the field of acromegaly.

## Data availability statement

The original contributions presented in the study are included in the article/Supplementary material, further inquiries can be directed to the corresponding authors.

## Author contributions

MW and YZ conceptualized the article and revised the draft. SP collected and analyzed the data and wrote the manuscript. QL collected and analyzed the data. YT, BH, and ZL prepared the figures. ML and JL prepared the tables. YZ and MW were the guarantors of the overall content. All authors approved the final version of the manuscript and agreed to be accountable for all the specifications of the study.

## Funding

This study was supported by the National Natural Science Foundation of China (82102581, 82270930, and 81873643), the National Postdoctoral Science Foundation of China (2021M693562), the Provincial Natural Science Foundation of Hunan (2019JJ40517 and 2022JJ40843), the Provincial Outstanding Postdoctoral Innovative Talents Program of Hunan (2021RC2020), the Foundation of Hunan Provincial Science and Technology Department (2021ZK4218), the Young Investigator Grant of Xiangya Hospital, Central South University (2020Q14), the FuQing Postdoc Program of Xiangya Hospital, Central South University (176), the Fund of Reform and Practice of Ideological and Political in Xiangya Hospital, Central South University (36, 40), the Teaching Reform Project of Hunan Province Regular Universities (HNJG-2021-0313), and the Hunan Provincial Degree and Postgraduate Teaching Reform Project (2021JGYB033).

## Conflict of interest

The authors declare that the research was conducted in the absence of any commercial or financial relationships that could be construed as a potential conflict of interest.

## Publisher’s note

All claims expressed in this article are solely those of the authors and do not necessarily represent those of their affiliated organizations, or those of the publisher, the editors and the reviewers. Any product that may be evaluated in this article, or claim that may be made by its manufacturer, is not guaranteed or endorsed by the publisher.

## References

[ref1] Ben-Shlomo A. Deng N. Ding E. Yamamoto M. Mamelak A. Chesnokova V.. (2020). DNA damage and growth hormone hypersecretion in pituitary somatotroph adenomas. J. Clin. Invest. 130, 5738–5755. doi: 10.1172/JCI138540 32673291 PMC7598090

[ref2] Biermasz N. R. Pereira A. M. Smit J. W. A. Romijn J. A. Roelfsema F. (2005). Morbidity after long-term remission for acromegaly: persisting joint-related complaints cause reduced quality of life. J. Clin. Endocrinol. Metabol. 90, 2731–2739. doi: 10.1210/jc.2004-2297 15741257

[ref3] Casanueva F. F. Barkan A. L. Buchfelder M. Klibanski A. Laws E. R. Loeffler J. S.. (2017). Criteria for the definition of pituitary tumor centers of excellence (PTCOE): a pituitary society statement. Pituitary 20, 489–498. doi: 10.1007/s11102-017-0838-2 28884415 PMC5606938

[ref4] Chen C. (2017). Science mapping: a systematic review of the literature. J. Data Inform. Sci. 2, 1–40. doi: 10.1515/jdis-2017-0006

[ref5] Chen C. Song M. (2019). Visualizing a field of research: a methodology of systematic scientometric reviews. PLoS One 14:e0223994. doi: 10.1371/journal.pone.0223994 31671124 PMC6822756

[ref6] Claessen K. M. J. A. Mazziotti G. Biermasz N. R. Giustina A. (2016). Bone and joint disorders in acromegaly. Neuroendocrinology 103, 86–95. doi: 10.1159/000375450 25633971

[ref7] Colao A. Ferone D. Marzullo P. Lombardi G. (2004). Systemic complications of acromegaly: epidemiology, pathogenesis, and management. Endocr. Rev. 25, 102–152. doi: 10.1210/er.2002-0022 14769829

[ref8] Colao A. Grasso L. F. S. Giustina A. Melmed S. Chanson P. Pereira A. M.. (2019). Acromegaly. Nat. Rev. Dis. Primers. 5:20. doi: 10.1038/s41572-019-0071-6 30899019

[ref9] Coopmans E. C. Andela C. D. Claessen K. M. J. A. Biermasz N. R. (2022). Evaluating the impact of acromegaly on quality of life. Endocrinol. Metab. Clin. N. Am. 51, 709–725. doi: 10.1016/j.ecl.2022.04.004 36244688

[ref10] Crisafulli S. Luxi N. Sultana J. Fontana A. Spagnolo F. Giuffrida G.. (2021). Global epidemiology of acromegaly: a systematic review and meta-analysis. Eur. J. Endocrinol. 185, 251–263. doi: 10.1530/EJE-21-0216 34061771

[ref11] Cuevas-Ramos D. Carmichael J. D. Cooper O. Bonert V. S. Gertych A. Mamelak A. N.. (2015). A structural and functional acromegaly classification. J. Clin. Endocrinol. Metab. 100:122. doi: 10.1210/jc.2014-2468 25250634 PMC4283008

[ref12] Dekkers O. M. Biermasz N. R. Pereira A. M. Romijn J. A. Vandenbroucke J. P. (2008). Mortality in acromegaly: a meta analysis. J. Clin. Endocrinol. Metab. 93, 61–67. doi: 10.1210/jc.2007-1191 17971431

[ref13] Fleseriu M. Fogelfeld L. Gordon M. B. Sisco J. Crosby R. D. Ludlam W. H.. (2020). An evaluation of the acromegaly treatment satisfaction questionnaire (Acro-TSQ) in adult patients with acromegaly, including correlations with other patient-reported outcome measures: data from two large multicenter international studies. Pituitary 23, 347–358. doi: 10.1007/s11102-020-01038-y 32221764 PMC7316852

[ref14] Fleseriu M. Langlois F. Lim D. S. T. Varlamov E. V. Melmed S. (2022). Acromegaly: pathogenesis, diagnosis, and management. Lancet Diabet. Endocrinol. 10, 804–826. doi: 10.1016/S2213-8587(22)00244-3 36209758

[ref15] Gadelha M. R. Kasuki L. Lim D. S. T. Fleseriu M. (2019). Systemic complications of acromegaly and the impact of the current treatment landscape: an update. Endocr. Rev. 40, 268–332. doi: 10.1210/er.2018-00115 30184064

[ref16] Ghazi A. A. Amirbaigloo A. Dezfooli A. A. Saadat N. Ghazi S. Pourafkari M.. (2013). Ectopic acromegaly due to growth hormone releasing hormone. Endocrine 43, 293–302. doi: 10.1007/s12020-012-9790-0 22983831 PMC3553305

[ref17] Giustina A. Barkan A. Beckers A. Biermasz N. Biller B. M. K. Boguszewski C.. (2020a). A consensus on the diagnosis and treatment of acromegaly comorbidities: an update. J. Clin. Endocrinol. Metab. 105:dgz096. doi: 10.1210/clinem/dgz096 31606735

[ref18] Giustina A. Barkan A. Casanueva F. F. Cavagnini F. Frohman L. Ho K.. (2000). Criteria for cure of acromegaly: a consensus statement. J. Clin. Endocrinol. Metab. 85, 526–529. doi: 10.1210/jc.85.2.526 10690849

[ref19] Giustina A. Barkhoudarian G. Beckers A. Ben-Shlomo A. Biermasz N. Biller B.. (2020b). Multidisciplinary management of acromegaly: a consensus. Rev. Endocr. Metab. Disord. 21, 667–678. doi: 10.1007/s11154-020-09588-z 32914330 PMC7942783

[ref20] Giustina A. Chanson P. Bronstein M. D. Klibanski A. Lamberts S. Casanueva F. F.. (2010). A consensus on criteria for cure of acromegaly. J. Clin. Endocrinol. Metab. 95, 3141–3148. doi: 10.1210/jc.2009-2670 20410227

[ref21] Giustina A. Mazziotti G. Canalis E. (2008). Growth hormone, insulin-like growth factors, and the skeleton. Endocr. Rev. 29, 535–559. doi: 10.1210/er.2007-0036 18436706 PMC2726838

[ref22] Hannah-Shmouni F. Trivellin G. Stratakis C. A. (2016). Genetics of gigantism and acromegaly. Growth Hormon. IGF Res. 30–31, 37–41. doi: 10.1016/j.ghir.2016.08.002 PMC5154831 27657986

[ref23] Holdaway I. M. (2004). Treatment of acromegaly. Horm. Res. 62, 79–92. doi: 10.1159/000080505 15539805

[ref24] Katznelson L. Laws E. R. Melmed S. Molitch M. E. Murad M. H. Utz A.. (2014). Acromegaly: an endocrine society clinical practice guideline. J. Clin. Endocrinol. Metab. 99, 3933–3951. doi: 10.1210/jc.2014-2700 25356808

[ref25] Marie P. (1886). Sur deux cas d’acromégalie; hypertrophie singulière non congénitale des extrémités supérieures, Inférieures et Céphalique. ed. Alcan F. (Paris: Rev Med), 297–333.

[ref26] Melmed S. (2006). Medical progress: acromegaly. N. Engl. J. Med. 355, 2558–2573. doi: 10.1056/NEJMra062453 17167139

[ref27] Melmed S. (2020). Pituitary-tumor endocrinopathies. N. Engl. J. Med. 382, 937–950. doi: 10.1056/NEJMra1810772 32130815

[ref28] Melmed S. Bronstein M. D. Chanson P. Klibanski A. Casanueva F. F. Wass J. A. H.. (2018). A consensus statement on acromegaly therapeutic outcomes. Nat. Rev. Endocrinol. 14, 552–561. doi: 10.1038/s41574-018-0058-5 30050156 PMC7136157

[ref29] Melmed S. Colao A. Barkan A. Molitch M. Grossman A. B. Kleinberg D.. (2009). Guidelines for acromegaly management: an update. J. Clin. Endocrinol. Metab. 94, 1509–1517. doi: 10.1210/jc.2008-2421 19208732

[ref30] Melmed S. Kaiser U. B. Lopes M. B. Bertherat J. Syro L. V. Raverot G.. (2022). Clinical biology of the pituitary adenoma. Endocr. Rev. 43, 1003–1037. doi: 10.1210/endrev/bnac010 35395078 PMC9695123

[ref31] Mizera L. Elbaum M. Daroszewski J. Bolanowski M. (2018). Cardiovascular complications of acromegaly. Acta Endocrinol. 14, 365–374. doi: 10.4183/aeb.2018.365 PMC6525769 31149285

[ref32] Orme S. M. McNally R. J. Cartwright R. A. Belchetz P. E. (1998). Mortality and cancer incidence in acromegaly: a retrospective cohort study. United Kingdom acromegaly study group. J. Clin. Endocrinol. Metab. 83, 2730–2734. doi: 10.1210/jc.83.8.2730 9709939

[ref33] Paunkov A. Chartoumpekis D. V. Ziros P. G. Sykiotis G. P. (2019). A bibliometric review of the Keap1/Nrf2 pathway and its related antioxidant compounds. Antioxidants 8:E353. doi: 10.3390/antiox8090353 PMC6769514 31480567

[ref34] Petrossians P. Daly A. F. Natchev E. Maione L. Blijdorp K. Sahnoun-Fathallah M.. (2017). Acromegaly at diagnosis in 3173 patients from the Liège acromegaly survey (LAS) database. Endocr. Relat. Cancer 24, 505–518. doi: 10.1530/ERC-17-0253 28733467 PMC5574208

[ref35] Pivonello R. Auriemma R. S. Delli Veneri A. Dassie F. Lorusso R. Ragonese M.. (2022). Global psychological assessment with the evaluation of life and sleep quality, sexual and cognitive function in a large number of patients with acromegaly: a cross-sectional study. Eur. J. Endocrinol. 187, 823–845. doi: 10.1530/EJE-22-0263 36165745 PMC9782455

[ref36] Potorac I. Bonneville J.-F. Daly A. F. de Herder W. Fainstein-Day P. Chanson P.. (2022). Pituitary MRI features in acromegaly resulting from ectopic GHRH secretion from a neuroendocrine tumor: analysis of 30 cases. J. Clin. Endocrinol. Metab. 107, E3313–E3320. doi: 10.1210/clinem/dgac274 35512251

[ref37] Trainer P. J. Drake W. M. Katznelson L. Freda P. U. Herman-Bonert V. van der Lely A. J.. (2000). Treatment of acromegaly with the growth hormone-receptor antagonist pegvisomant. N. Engl. J. Med. 342, 1171–1177. doi: 10.1056/NEJM200004203421604 10770982

[ref38] Wassenaar M. J. E. Biermasz N. R. Hamdy N. A. T. Zillikens M. C. Meurs J. B. J. Van Rivadeneira F.. (2011). High prevalence of vertebral fractures despite normal bone mineral density in patients with long-term controlled acromegaly. Eur. J. Endocrinol. 164, 475–483. doi: 10.1530/EJE-10-1005 21257726

[ref39] Zhang J. Song L. Xu L. Fan Y. Wang T. Tian W.. (2021). Knowledge domain and emerging trends in Ferroptosis research: a bibliometric and knowledge-map analysis. Front. Oncol. 11:686726. doi: 10.3389/fonc.2021.686726 34150654 PMC8209495

