# Supplementary material for: A worldwide bibliometric analysis of acromegaly in the past two decades: 1999–2022
Source: Front Neurosci. 2023 Jul 5;17:1187820. doi: 10.3389/fnins.2023.1187820 (PMC10354554; doi:10.3389/fnins.2023.1187820)
Supplement: Supplementary file 1 [file Data_Sheet_1.docx]

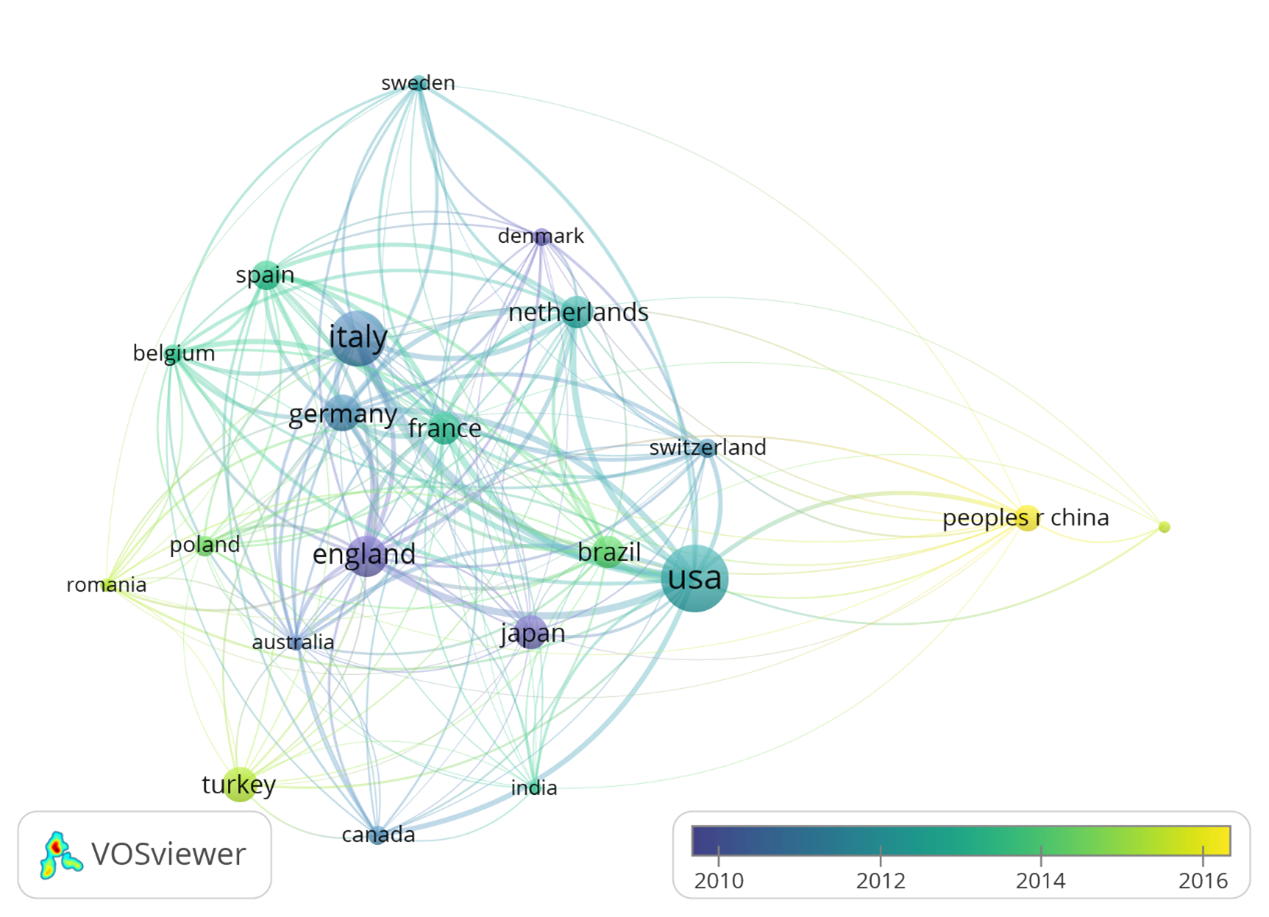


Supplementary figure 1 The overlay map of the average year of country/region of acromegaly. The node size represents co-occurrence frequency, the links represent co-occurrence relationship, and the different colors indicate the relevant year of publication. Yellow keywords came later than blue keywords. The minimum number of publications is 50.


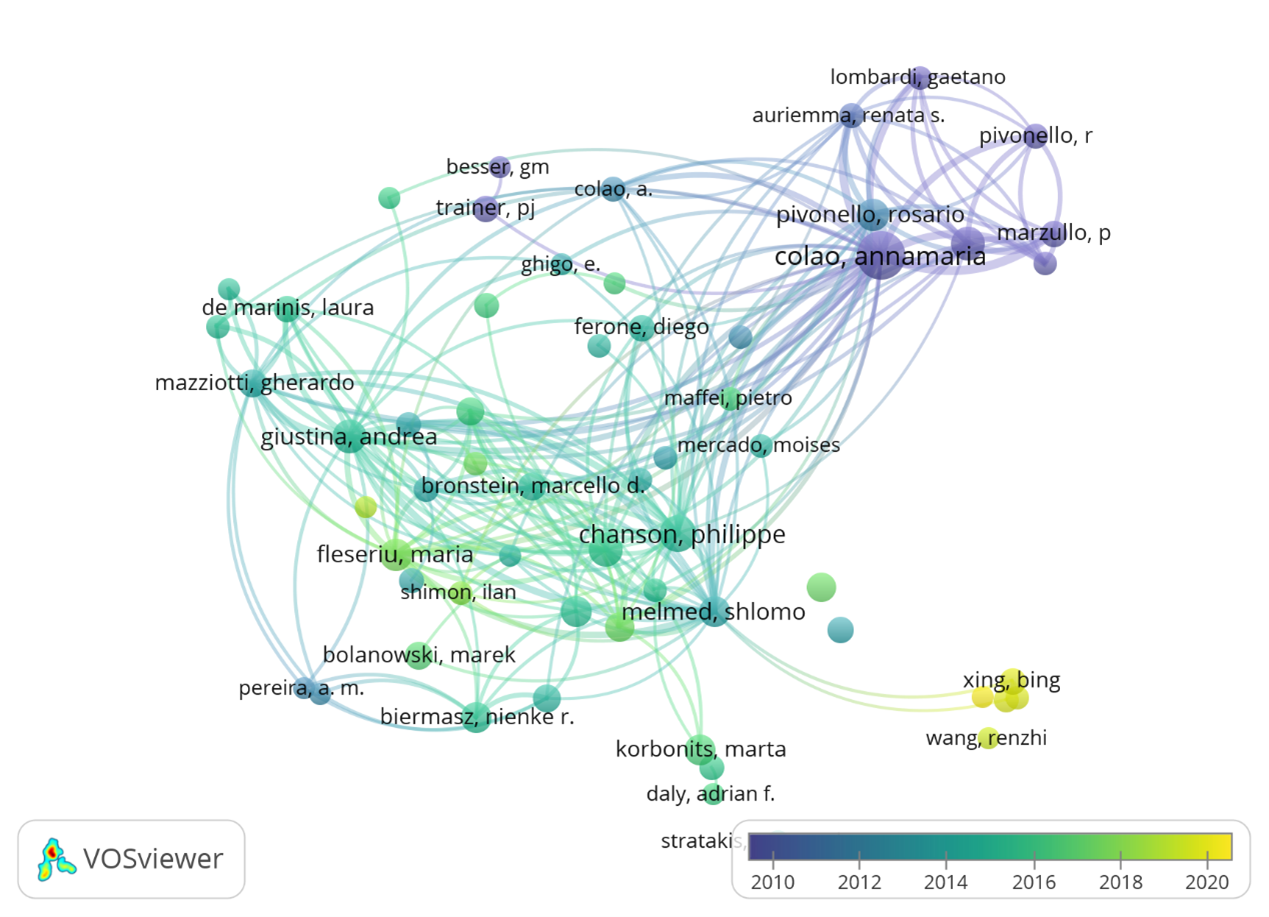


Supplementary figure 2 The visual map of the co-cited Author of acromegaly. The node size represents co-occurrence frequency, the links represent co-occurrence relationship, and the different colors indicate the relevant year of publication. Yellow keywords came later than blue keywords. The minimum number of publications is 20.


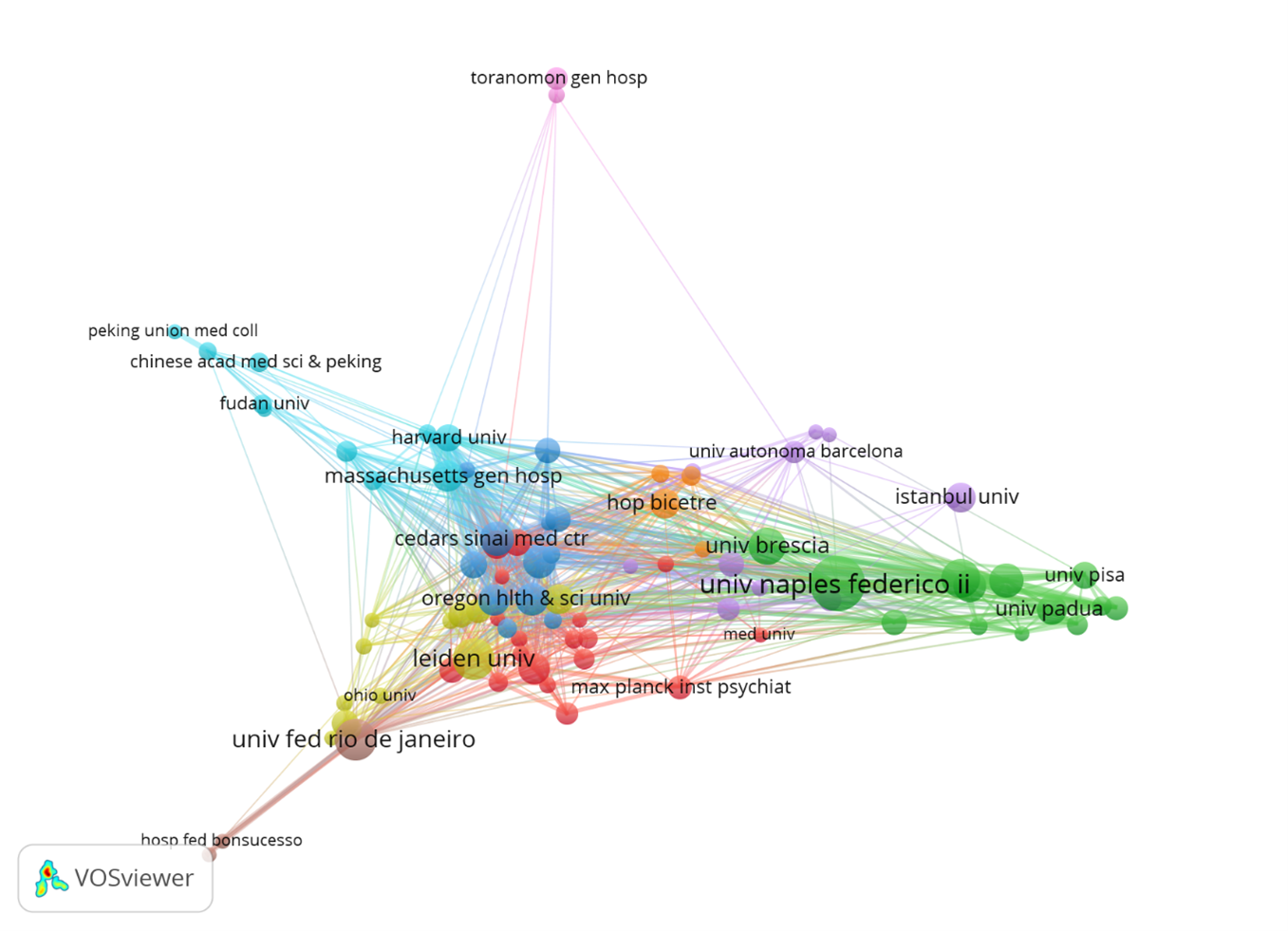


Supplementary figure 3 The visual map of institutions in acromegaly research. The node size represents co-occurrence frequency, and the links represent co-occurrence relationship. The node color represents its affiliation to different co-occurrence clusters. Using different colors, 85 out of the 3587 institutions appeared more than 20 times, and were separated into nine clusters.


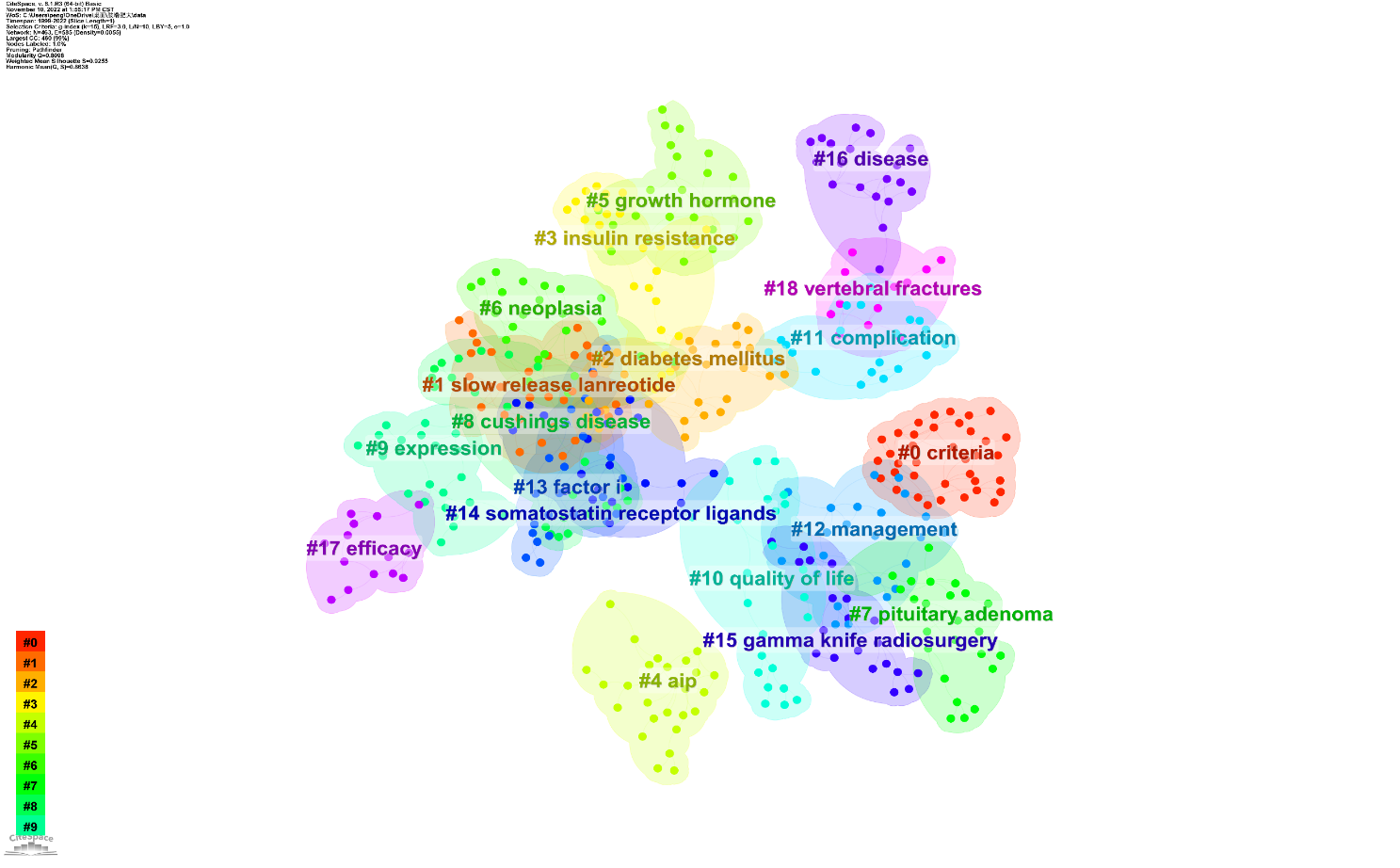


Supplementary figure 4 The keywords clustering. Cluster labels were extracted from title by LLR, and the color represents its affiliation to different keywords clusters. The smaller the number, the larger the cluster, and #0 is the largest cluster.
